# Contemporary selection on MHC genes in a free‐living ruminant population

**DOI:** 10.1111/ele.13957

**Published:** 2022-01-20

**Authors:** Wei Huang, Kara L. Dicks, Jarrod D. Hadfield, Susan E. Johnston, Keith T. Ballingall, Josephine M. Pemberton

**Affiliations:** ^1^ Institute of Evolutionary Biology School of Biological Sciences University of Edinburgh Edinburgh UK; ^2^ Royal Zoological Society of Scotland Edinburgh UK; ^3^ Moredun Research Institute Edinburgh UK

**Keywords:** MHC, selection, soay sheep

## Abstract

Genes within the major histocompatibility complex (MHC) are the most variable identified in vertebrates. Pathogen‐mediated selection is believed to be the main force maintaining MHC diversity. However, relatively few studies have demonstrated contemporary selection on MHC genes. Here, we examine associations between MHC variation and several fitness measurements including total fitness and five fitness components, in 3400 wild Soay sheep *(Ovis aries*) monitored between 1989 and 2012. In terms of total fitness, measured as lifetime breeding success of all individuals born, we found haplotypes named C and D were associated with decreased and increased male total fitness respectively. In terms of fitness components, juvenile survival was associated with haplotype divergence while individual haplotypes (C, D and F) were associated with adult fitness components. Consistent with the increased male total fitness, the rarest haplotype D has increased in frequency throughout the study period more than expected under neutral expectations. Our results demonstrate that contemporary natural selection is acting on MHC class II genes in Soay sheep and that the mode of selection on specific fitness components can be different mode from selection on total fitness.

## Graphical abstract

The major histocompatibility complex (MHC) is one of the most variable gene families in vertebrates. Parasite‐mediated selection is believed to be the main force maintaining diversity at MHC genes, but demonstrating contemporary selection on MHC alleles in natural populations is not trivial. Here, we use haplotypes of expressed MHC class II genes in a large sample of individually monitored wild sheep to examine associations between MHC and fitness measurements and investigate change in MHC haplotype frequency over two decades. With this large data set, we demonstrate contemporary selection on MHC genes and highlight highlights the importance of investigating frequency change of MHC haplotype.

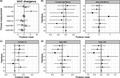

## INTRODUCTION

The vertebrate immune system has evolved to protect the host from infection through a variety of innate and adaptive immune mechanisms. Many of the proteins that control the specificity of immune function are the products of genes that are among the most diverse within vertebrate populations. Such diversity is believed to have evolved in response to pathogen diversity. Investigating the mechanisms shaping the diversity of immune genes has been a major focus in evolutionary biology (Barreiro & Quintana‐Murci, 2010). Genes located within the major histocompatibility complex (MHC) encode proteins that play important roles in the vertebrate immune system and have been intensively studied across many species, including model organisms (Acevedo‐Whitehouse & Cunningham, 2006; Bernatchez & Landry, 2003; Kaufman, 2018; Piertney & Oliver, 2006; Radwan et al., 2020). MHC molecules, the products of MHC class I and class II genes, present peptide fragments to antigen‐specific receptors on T cells to induce adaptive immune responses. MHC class I molecules present peptides mainly derived from intracellular pathogens such as viruses to CD8+ T cells, while MHC class II molecules present peptides mainly derived from extracellular pathogens such as bacteria and parasites to CD4+ T cells (Garrigan & Hedrick, 2003).

MHC class I and II genes are the most polymorphic loci identified in vertebrate populations with hundreds of alleles described in many species (Apanius et al., 1997; Maccari et al., 2017). Although MHC‐based sexual selection and genetic drift can maintain MHC diversity, pathogen‐mediated balancing selection is thought to be the principal driver maintaining such high levels of allelic diversity (Bolnick & Stutz, 2017; Oliver et al., 2009; Oppelt et al., 2010; Radwan et al., 2020; Westerdahl et al., 2012). However, the specific selection mechanisms which act to maintain high levels of MHC allelic diversity are not clear (Bernatchez & Landry, 2003; Jordan & Bruford, 1998; Spurgin & Richardson, 2010). Three models have been proposed to explain how PMS acts to maintain such diversity:

1. *Heterozygote advantage*: individuals which are more heterozygous across the MHC can respond to a greater variety of pathogens. As a result, variation at MHC loci is selected and maintained (Takahata & Nei, 1990). In addition, *divergent allele advantage* has been proposed as an extension to heterozygote advantage. Here, individuals with a greater functional divergence between alleles should have a selective advantage because their MHC molecules can bind a broader range of antigens (Eizaguirre et al., 2012a; Pierini & Lenz, 2018; Wakeland et al., 1990).
2. *Rare*‐*allele advantage*: there is strong selection on pathogens to evade immune protection provided by the most common host MHC alleles. Therefore, a rare allele which confers resistance to the most selective pathogen will have a selective advantage (Eizaguirre et al., 2012b). However, the advantage disappears as the frequency of a protective MHC allele increases and is absent at equilibrium (Lenz, 2018; Phillips et al., 2018). The arms race between hosts and pathogens sets up a cyclical process causing MHC alleles and pathogens to fluctuate, meaning that MHC diversity is maintained by negative frequency‐dependent selection (Borghans et al., 2004; Ejsmond & Radwan, 2015; Hughes & Nei, 1988). It should be noted that since rare alleles appear most frequently as heterozygotes, this can manifest as heterozygote advantage for specific alleles under some circumstances.
3. *Fluctuating selection*: a changing environment results in variation in the abundance of different pathogens in space and time, which generates directional selection in different host subpopulations and/or at different time points. Thus, different alleles will be favoured in different subpopulations or at different times, meaning MHC diversity is maintained as a consequence (Hedrick, 2002; Hill et al., 1991). Unlike rare‐allele advantage the change in selection coefficients in time and/or space is not driven by coevolution in the pathogens.

To test each selection model we can examine association between MHC variation and fitness measurements, different patterns of associations can be explained as a result of different selection mechanisms (Piertney & Oliver, 2006; Spurgin & Richardson, 2010; Sutton et al., 2011). Under heterozygote advantage, we predict a positive association between MHC heterozygosity and fitness. Under rare‐allele advantage, if MHC alleles are not in equilibrium, we may detect selection favouring specific rare alleles and probably also disfavouring specific common alleles. Under fluctuating selection, we would also observe specific alleles being favoured or disfavoured by selection. But unlike rare‐allele advantage, this should occur regardless of their frequency. However, it is hard to distinguish rare‐allele advantage and fluctuating selection by examining MHC‐fitness associations unless investigating the same alleles in parallel populations (Spurgin & Richardson, 2010). A number of studies have tested associations between fitness and MHC variation in wild populations, with mixed results. First, some MHC studies in wild populations have found a positive association between MHC heterozygosity and fitness. For instance the number of MHC alleles was found to be positively associated with increased apparent survival (return rate from initial year of capture) in common yellowthroats *(Geothlypis trichas)* (Dunn et al., 2013). Similarly, Banks et al. (2010) found an association between MHC heterozygosity and survival in a natural population of mountain brushtail possums *(Trichosurus cunninghami)*. Second, some studies have reported associations between fitness and specific MHC alleles or supertypes (clusters of MHC alleles with similar physicochemical properties at their antigen‐binding sites). For example a study of great tits *(Parus major)* using mark‐recapture data identified three MHC supertypes associated with fitness components, one with increased survival, one with increased lifetime reproductive success and another with decreased lifetime reproductive success (Sepil et al., 2013). Kloch et al. (2013) also found that two MHC alleles from different phylogenetic clusters were associated with winter survival in root voles *(Microtus oeconomus)*. Finally, there are some studies where no associations are detected. In collared flycatchers (*Ficedula albicollis*), no association between MHC variation and lifetime reproductive success was found (Radwan et al., 2012).

A complication in studies of fitness association is that associations may differ between individuals of different sex, age or nutritional status, due to variation in the pathogen community that they are exposed to. For example in a population of black‐legged kittiwake (*Rissa tridactyla*), the association between MHC diversity and fitness was only found in second‐hatched female chicks suggesting sex and hatching order could modulate MHC‐fitness association (Pineaux et al., 2020). In a previous study of Seychelles warbler (*Acrocephalus sechellensis*), a positive association with MHC diversity was only found in juvenile survival but not in adult survival (Brouwer et al., 2010). However, the impact of age on MHC‐fitness associations has been rarely studied because of the limitation of study length and scale in wild populations. Thus, longitudinal studies with fitness measurement through all life stages of study individuals are required to study MHC‐dependent fitness effects.

Apart from the difficulty in measuring fitness to study selection on MHC genes, many previous studies of selection on MHC genes are limited by the depth and quality of the genetic data available. Within a typical vertebrate MHC region, multiple closely linked and duplicated genes are present which are inherited together as a haplotype. Although with the development of next‐generation sequencing (NGS) technologies, we are now able to cost‐effectively genotype large numbers of samples (Babik, 2010; Babik et al., 2009). However, it is still difficult to characterise MHC diversity in species with complex MHC structure such as passerine birds.

Lastly, statistical analyses of selection on MHC variation could be more precise for controlling genetic confounders than is often deployed. When examining contemporary selection on MHC genes in long‐term individual‐based studies, which include a lot of genetically related individuals (Brouwer et al., 2010; Sepil et al., 2013), it is possible that MHC similarity may confound with genetic relatedness such that an MHC effect could be due to variation elsewhere in the genome rather than the MHC itself. Therefore, MHC studies should be conducted in animal models when possible. In addition, given the ubiquity of inbreeding depression, any analysis of selection should include an estimate of individual inbreeding to ensure MHC effects are not confounded with genome‐wide inbreeding effects (Lenz et al., 2013; Thoss et al., 2011).

Here, we present the first investigation of contemporary selection acting on the MHC in a free‐living population that overcomes the issues raised above. The Soay sheep *(Ovis aries*) population on the island of Hirta, St Kilda is one of the most intensively studied wild animal populations in the world (Clutton‐Brock & Pemberton, 2004). Since 1985, taking advantage of the island landscape, nearly all individual Soay sheep living in the Village Bay study area have been followed from birth, through all breeding attempts, until death. In addition, using a combination of observation and SNP genotypes, a multigenerational pedigree for the population has been assembled which includes annual and lifetime measures of fitness for both sexes (Bérénos et al., 2015). Previous work characterised diversity at the MHC class II *DRB1* gene using an internal microsatellite and identified associations between MHC genotype, fitness and parasite infection (Paterson, 1998; Paterson et al., 1998). However, the only fitness measure studied was juvenile survival. Another study using the same genetic data set of MHC‐linked microsatellite alleles found higher temporal and spatial variation in these loci than in putatively neutral microsatellite loci in other parts of the genome, suggesting fluctuating selection on MHC genes (Charbonnel & Pemberton, 2005). Recently, using sequencing‐based genotyping of functional MHC class II DR and DQ loci we have shown that eight MHC haplotypes are segregating in the study population (Dicks et al., 2019) and MHC class II diplotypes were characterised for 5349 individuals sampled between 1989 and 2012 (Dicks et al., 2021). Here, we use this data to investigate associations between MHC variation and fitness. We test for associations between (1) MHC variation and total fitness, defined as lifetime breeding success of each individual and (2) MHC variation and five fitness components. We fit MHC variation including divergence, heterozygosity and individual haplotypes within quantitative genetic models in order to distinguish the effect of MHC haplotypes from genome‐wide effects. Finally (3), we perform gene‐drop simulations to examine whether haplotype frequencies have changed more than expected from a neutral process.

## METHODS

### Study system

Soay sheep have lived virtually unmanaged on the island of Soay, in the St. Kilda archipelago, for thousands of years. In 1932, 107 Soay sheep were introduced to the larger neighbouring island of Hirta and have been living there unmanaged since. A previous study demonstrated that an introgression event between Soay sheep and a more modern breed occurred approximately 150 years ago (Feulner et al., 2013). From 1985, a long‐term study has been conducted on the sheep resident in the Village Bay area of Hirta to investigate ecological and evolutionary questions (Clutton‐Brock & Pemberton, 2004). As an island isolate, the population has low Ne and low genetic diversity compared with other sheep breeds (Kijas et al., 2012) and is quite inbred (Stoffel et al., 2021). The vast majority of lambs are born in April or May of each year. At their first live capture, lambs are ear‐tagged to enable life‐long identification and discs of ear tissue removed in the tagging process are used for DNA extraction. Parentage is inferred for each individual using a subset of 315 unlinked SNPs derived from the Illumina Ovine 50K SNP array, on which most individuals alive since 1990 have been genotyped (Bérénos et al., 2014). In addition, the R package Sequoia (Huisman, 2017) was used to cluster half‐siblings which share a parent that has not been genotyped. In cases where no SNP genotypes were available, a small number of parentage inferences were made using field observations (for mothers) or a previous microsatellite genotype approach (Morrissey et al., 2012). The pedigree used here includes 7447 individuals, with 7014 paternal links and 6229 maternal links. This study investigated associations between MHC variation and fitness measurements including total fitness and five fitness components defined as follows:

- Total fitness, the number of offspring an individual produced over its lifetime. This measure includes all sheep born, regardless of whether they survived and bred (many did not);
- Juvenile survival, a binary variable indicating whether a lamb survived from birth until May 1st of the following year.
- Adult annual survival, a binary variable indicating whether an adult survived or not in a given calendar year; an adult is an animal that survived to age 1.
- Adult life span, the age of an adult when it died;
- Adult annual breeding success, the number of offspring an adult that survived the juvenile period produced in a given calendar year;
- Adult lifetime breeding success, the number of offspring an adult that survived the juvenile period produced over its lifetime;

### MHC class IIa haplotyping

**FIGURE 1:**
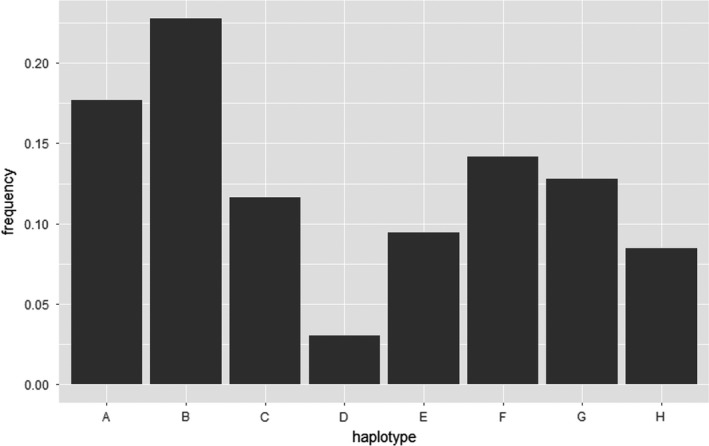
The frequency of each MHC haplotype in the sample of Soay sheep used in the study (*N* = 3440 individuals)

The genetic data used in this study were obtained from previous studies (Dicks et al., 2019, 2021). In these studies, the seven expressed loci (DRB1, *DQA1*, *DQA2*, *DQA2*‐*like*, *DQB1*, *DQB2* and *DQB2*‐*like*) within the MHC class IIa region were characterised in 118 selected Soay sheep using genotyping‐by‐sequencing. A total of eight MHC haplotypes were identified and named A to H. These haplotypes were confirmed by genotyping‐by‐sequencing the same loci in an additional 94 individuals with no new haplotypes found. A panel of 13 SNPs located in the region of MHC class IIa haplotypes was then selected to impute the eight MHC class IIa haplotypes in 5951 Soay sheep genotyped using Kompetitive Allele‐specific PCR (KASP). This panel included 11 SNPs known to be variable in Soays based on data from the Illumina Ovine infinium HD chip and two other SNPs identified within the *DQA1* gene (Dicks et al., 2021). After quality control, the diplotypes of 5349 individuals that lived in the study area between 1985 and 2012 were identified. As cohort effects were fitted in the statistical analyses, we excluded animals born before 1989 since too few were genotyped to be representative of their birth cohort. We also removed the cohort born in 2010 which had a high genotyping failure rate in the KASP genotyping assay, all animals which died as foetuses and any individuals subjected to experimental treatments which may have affected their survival or breeding performance (e.g. anthelmintic boluses). After these exclusions, a total of 3440 individuals (1750 males and 1650 females) remained for statistical modelling of total fitness and juvenile survival (Table 1). The frequency of the rarest haplotype (D) was 3.02%. With the exception of animals homozygous for haplotype D (*N* = 5), moderate numbers of individuals (*N* > 20) of each diplotype are represented (Figure 1 and Supplementary 2). For each individual successfully diplotyped, MHC divergence was measured as the proportion of the amino acid sequence that differed between the two MHC haplotypes (p‐distance) (Henikoff, 1996) (Note S1, Figure S2).

**TABLE 1:** Fixed and random effects fitted in null model and sample sizes, for each fitness measurement. Sample sizes are shown by number of individuals (records)

Fitness measurement	Fixed effects			Random effects			Sample sizes	
	Litter size	Age (years, quadratic)	Inbreeding (F_grm_)	Birth year	Year of fitness measure	ID	Female	Male
Total fitness			×	×			1650	1750
Juvenile survival	×		×	×			1650	1750
Adult annual survival		×	×	×	×	×	551 (3093)	529 (1610)
Adult lifetime breeding success			×	×			574	545
Adult annual breeding success		×	×	×	×	×	574 (3667)	545 (2155)
Adult lifetime breeding success			×	×			574	545

### Statistical analysis

We used generalised linear mixed models (GLMMs) to study the associations between MHC haplotypes and fitness measurements. The primary models were animal models that include a covariance structure proportional to the pedigree relatedness matrix, but we also repeated all tests without pedigree information. In null models, we fitted a set of fixed and random effects for each fitness measurement (Table 2) including genomic inbreeding (F_grm_) as a fixed effect, as previous studies demonstrated inbreeding depression in this population (Bérénos et al., 2016; Stoffel et al., 2021). None of the fitness measurements in our study had a normal distribution, so we used Markov chain Monte Carlo techniques implemented in MCMCglmm v 2.28 (Hadfield, 2010) to run models.

To examine whether there were associations between MHC variation and fitness measurements, we fitted MHC heterozygosity, MHC divergence and individual MHC haplotypes simultaneously into the null model. Each haplotype was fitted as dosage (0, 1 or 2 copies) as a fixed effect in all the models. We performed a Wald test using the posterior mean and the posterior covariance matrix to test whether MHC haplotypes explained variation in fitness. In these models, Haplotype H was treated as a reference so the results for individual haplotypes are relative to haplotype H and there are seven degrees of freedom. When a Wald test for haplotype differences was significant, we conducted an additional analysis (Note S2) comparing the estimate for each haplotype with the mean of the estimates for all other haplotypes, to identify individual haplotype effects.

Finally, we used models with different error distributions to combat the heterogeneity of ontogenies and distributions of fitness measurements (Note S3). We used probit regression to run a unisex model for juvenile survival. As the ontogenies and distributions of total fitness and adult fitness components were different between the two sexes, we used separate models for males and females. For both males and females, we used zero‐inflated Poisson models for total fitness, probit regression for adult annual survival and Poisson regression for adult life span and lifetime breeding success. As females can only have up to three offspring each year while males had between 0 and 24, we used multiple category probit (ordinal) regression for female annual breeding success and Poisson regression for male annual breeding success. All the models were run for 200,000 iterations in R v3.5.2 (R Core Team, 2021).

### *Gene*‐*drop analysis*

We performed gene‐drop simulations with a custom R library genedroppeR v 0.1.0 (code available at https://github.com/susjoh/genedroppeR) in R v3.5.2 (58) to simulate the expected frequency changes of MHC haplotypes under genetic drift given an identical pedigree structure. Hereafter, the first four birth cohorts (1989 to 1992) are defined as the ‘founder’ cohorts, whereas all subsequent cohorts are defined as ‘simulated’ cohorts (1993–2012). A total of 5000 simulations were conducted as follows. Individuals in the founder cohorts were assigned their observed MHC diplotypes; founder cohort individuals with unknown diplotypes were assigned one by either: (a) sampling a haplotype from each parent assuming Mendelian segregation if the parent was known; and/or (b) sampling a haplotype with the probability of the observed haplotype frequencies in the same birth cohort when the parent was unknown. In the simulated cohorts, each individual diplotype was sampled using approaches (a) and (b) above. By this method, simulated diplotypes were generated for each individual in the pedigree born after 1992. Using these data with the record of birth year and death year of each individual, we compared the observed and simulated changes in MHC haplotype frequency for the standing population (all the individuals living in a single year), using two approaches. First, we modelled the directional change in allele frequency using a linear regression with allele frequency as the response variable and year (1993 to 2012) as the predictor variable. If the observed slope fell within the top or bottom 2.5% of simulated slopes, the haplotype was deemed likely to have been subject to positive or negative directional selection respectively. Second, we modelled the cumulative change in allele frequency, defined as the sum of the absolute (i.e. positive) differences in allele frequency between consecutive years from 1993 to 2012. If this value fell within the top 2.5% of simulated slopes, the haplotype was deemed to have been subject to fluctuating selection. Alternatively, if the cumulative change fell within the bottom 2.5% of simulated slopes, the haplotype was deemed to have been subject to selection maintaining the haplotype at an equilibrium value.

## RESULTS

### Associations between MHC and fitness

Here we report the results of animal models, that is including the pedigree as a random effect.

**FIGURE 2:**
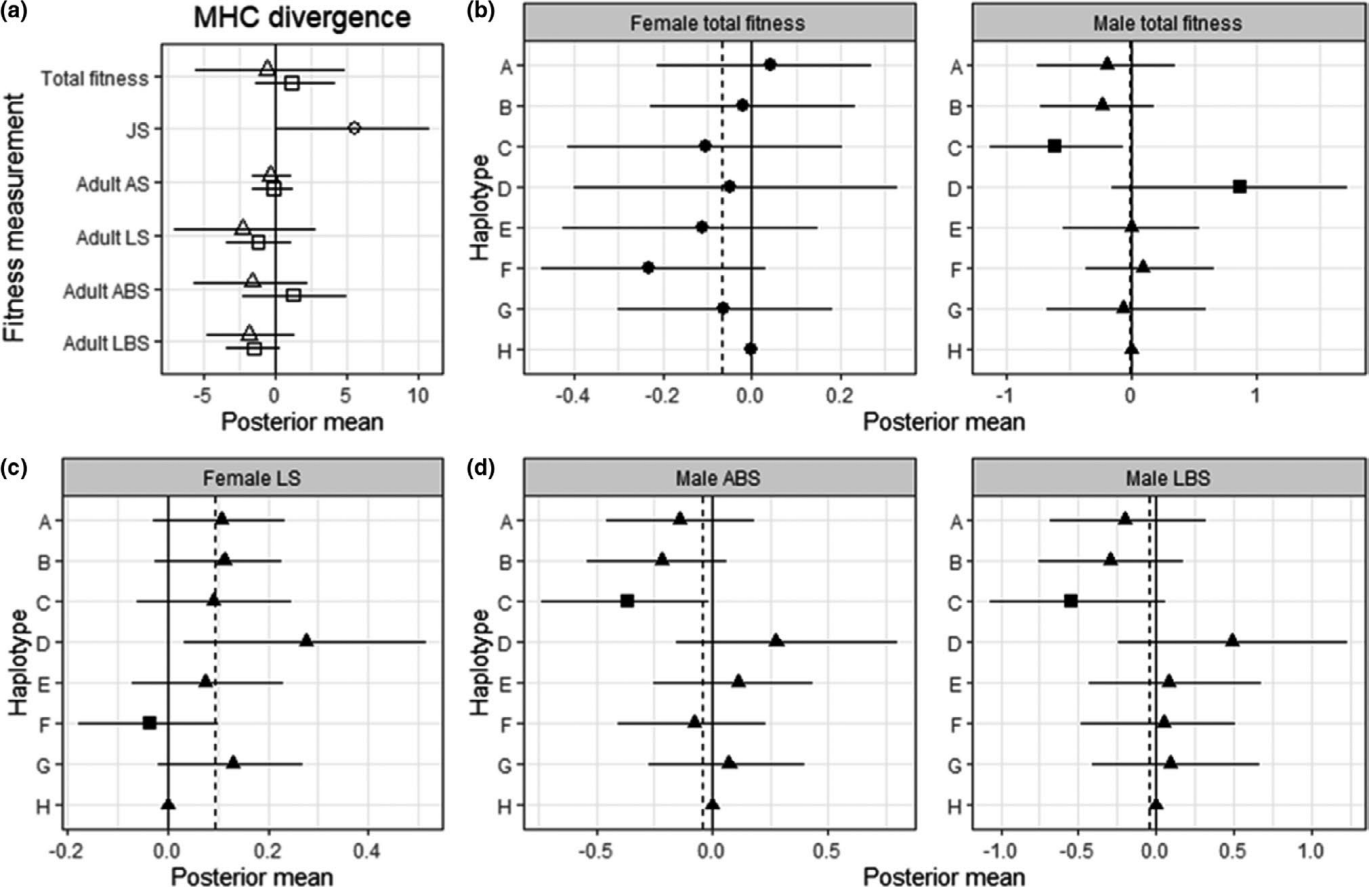
Associations between MHC and fitness measurements in Soay sheep derived from animal models. (a) Associations between MHC divergence and fitness measurements in juveniles (circle), females (squares) and males (triangles). Bars represent the 95% credibility intervals and those not overlapped with zero represent significant results. (b) Associations between MHC haplotypes and total fitness in females and males. The solid line represents the model intercept at haplotype H and posterior means and credible intervals for each haplotype are plotted relative to H. Dashed lines indicate the average posterior mean of all eight haplotypes. Circles indicate the Wald test was not significant, other symbols indicate it was significant. Haplotype effects that were significantly different from the mean are shown as squares with non‐significant ones show as triangles. (c and d) Associations between MHC haplotypes and adult fitness components in females (c) and males (d). Symbols and lines as for (b)

Total fitness: there was no association between MHC heterozygosity or MHC divergence and total fitness in either sex (Figure 2a). In the Wald test for haplotype effects significant associations were found for males, but not for females (Table 2). Haplotype C was associated with decreased male total fitness while haplotype D was associated with increased total fitness (Figure 2b).

**TABLE 2:** Results (*p* value) of Wald tests for animal models testing for differences between haplotypes in fitness measures (d.f. = 7)

Sex	Fitness measurement	*p* value
Both	Juvenile survival	0.13
Female	Total fitness	0.45
	Adult annual survival	0.073
	**Adult life span**	**0.028**
	Adult annual breeding success	0.92
	Adult lifetime breeding success	0.12
Male	**Total fitness**	**0.0034**
	Adult annual survival	0.39
	Adult life span	0.55
	**Adult annual breeding success**	**0.019**
	**Adult lifetime breeding success**	**0.039**

Bold number show significant results (*p* < 0.05).

Juvenile survival: we found a positive association between MHC divergence and juvenile survival (Figure 2a). Considering haplotype effects, the Wald test did not reveal significant associations (Table 2).

Adult annual survival: there was no association between MHC heterozygosity or MHC divergence and adult annual survival in either sex (Figure 2a). The Wald test for haplotype effects did not reveal significant associations (Table 2).

Adult lifespan: there was no association between MHC heterozygosity or MHC divergence and adult life span in either sex (Figure 2a). In the Wald test for haplotype effects a significant association was found for females, but not for males (Table 2). Haplotype F was associated with decreased adult female life span (Figure 2c).

Adult annual breeding success: there was no association between MHC heterozygosity or MHC divergence and annual breeding success in either sex (Figure 2a). In the Wald test for haplotype effects a significant association was found for males, but not for females (Table 2). Haplotype C was associated with decreased adult male annual breeding success (Figure 2d).

Adult lifetime breeding success: we found no association between MHC heterozygosity or MHC divergence and adult lifetime breeding success in either sex (Figure 2a). In the Wald test for haplotype effects a significant association was found for males, but not for females (Table 2). Haplotype C was associated with decreased adult male lifetime breeding success (Figure 2d).

The results obtained from models not including the pedigree were generally consistent with those above for associations between MHC heterozygosity/divergence and fitness measurements (Figures S8 and S10, Table S2). For associations between individual MHC haplotypes and fitness measurements, estimates were mostly similar, but there were minor variations in terms of significance of individual haplotypes (Table 2, Table S2). One difference was that we that found haplotype D was associated with increased female adult life span in models without pedigree fitted (Tables S9 and S10, Figure S7).

### *Gene*‐*drop analysis*

**FIGURE 3:**
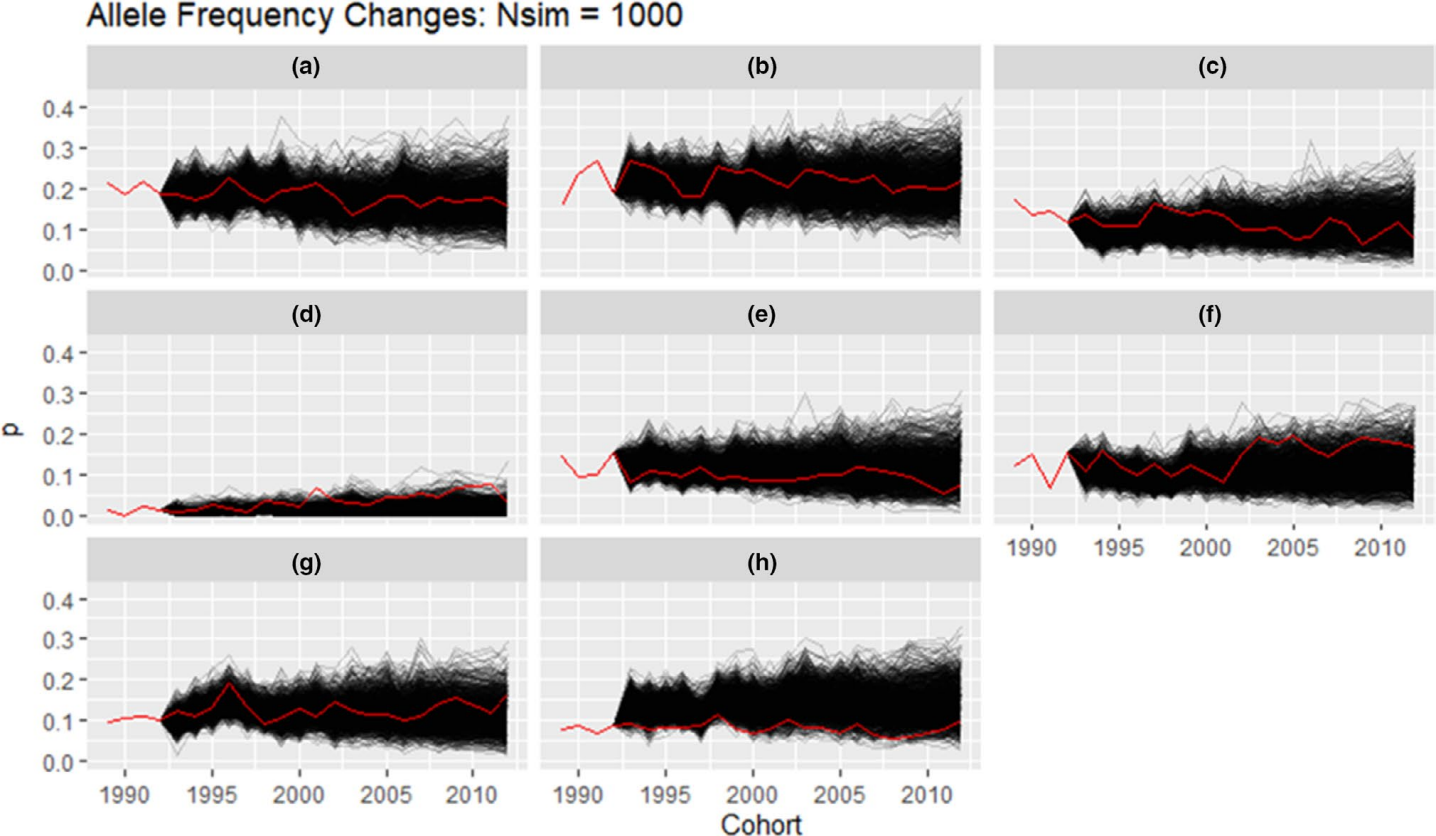
Results of the gene drop analysis for each haplotype. The black lines represent the result of simulations (*N* = 5000) and the red lines represent the observed frequency of each haplotype in the Soay sheep population over the same time interval

All observed haplotype frequency changes fell within the expected range due to drift alone, with the exception of haplotypes A, D and H (when compared to *N* = 5000 simulations; Figure 3). Haplotype D showed evidence of directional selection, with its frequency increasing more than expected due to chance between 1993 and 2012 (*p* = 0.0194; Table S11). Haplotypes A and H showed a much lower cumulative change than expected between 1993 and 2012 (*p* = 0.00040 and = 0.00038 respectively; Table S12), indicating balancing selection keeping allele frequencies at an equilibrium value.

## DISCUSSION

In this study, we investigated associations between MHC class II diversity (heterozygosity, divergence and specific haplotypes) and fitness measurements including total fitness and five fitness components in a sample of individually monitored free‐living ruminants using generalised linear mixed models. We fitted MHC divergence, MHC heterozygosity and individual MHC haplotype effects together to test whether there is heterozygote/divergent allele advantage, rare‐allele advantage or fluctuating selection. For all models, we included genetic relatedness to eliminate potential false‐positive results due to other genomic regions in common in related individuals and a genome inbreeding estimator to control for genome‐wide heterozygosity and the effect of inbreeding depression. We found a negative association between haplotype C and male total fitness and a positive association between haplotype D and male total fitness. We found MHC divergence was associated with increased juvenile survival. We also identified a negative association between haplotype C and adult male adult annual and lifetime breeding success and a negative association between haplotype F and adult female lifespan. Finally, we found that the rarest and the most divergent haplotype (Figure 1 and Figure S2), D, increased significantly in frequency, and the cumulative change of haplotype A and H more even than expected, over the 23‐year study period.

Our study illustrates that selection in specific fitness components may not be reflected in total fitness. We found an association with MHC divergence in juvenile survival, but did not observe this effect in total fitness or adult fitness components. These results are consistent with a previous study of Seychelles warbler that found a positive association between MHC diversity and juvenile but not adult survival (Brouwer et al., 2010). In Soay sheep, parasite infection intensity (measured as strongyle egg count) (Craig et al., 2006) and mortality is highest in the juvenile period (Clutton‐Brock & Pemberton, 2004) and parasite infection intensity is also negatively correlated with juvenile survival (Hayward et al., 2011). Therefore, lambs with divergent MHC haplotypes may be better able to respond to a wider range of pathogen antigens, increasing their chances of surviving the harsh juvenile period. However, as more MHC homozygote lambs die in their first year, selection for divergent MHC constitution may no longer be evident in adults due to selective disappearance. Alternatively, the lack of selection for MHC heterozygosity or divergence in adult sheep could also result from maturation of the immune system or a change in the parasite community (Craig et al., 2006; Sparks et al., 2018). In terms of specific haplotypes, we did not find any association between specific MHC haplotypes and juvenile survival, but we did detect associations with total fitness and adult fitness components which could be a reflection of the heterogeneity of parasite infection and the development of immunity to parasites acquired between the juvenile and adult periods. The associations between specific haplotypes and total fitness demonstrate signatures of directional selection which could be either rare‐allele advantage or fluctuating selection is in operation, similar to previous studies (Paterson et al., 1998; Sepil et al., 2013). However, we were not able to differentiate the two hypotheses using current data.

Although many studies in wild populations have identified associations between specific MHC alleles and fitness, few studies have observed an allele frequency change in response to selection (Biedrzycka et al., 2018; Westerdahl et al., 2004). For example a study of the great reed warbler (*Acrocephalus arundinaceus*) identified significantly higher variation in MHC allele frequencies between cohorts than at neutral loci, suggesting that fluctuating selection may be acting on MHC variation (Westerdahl et al., 2004). In a previous experimental study, MHC alleles providing resistance to the respective specific parasite increased in frequency in the next host generation of sticklebacks (*Gasterosteus aculeatus*) (Eizaguirre et al., 2012b). If MHC‐linked fitness effects are strong enough, we should be able to see changes in haplotype frequency over time in Soay sheep. In our study, we found haplotype C and D were associated with decreased and increased male total fitness respectively. In addition, the point estimates of haplotype C and D for female total fitness are, if anything, in the same direction (though not significant). Consistent with this, the gene drop analysis showed that haplotype D has increased significantly through the study period (*p* = 0.0194). However, the frequency of haplotype C did not change more than expected by chance. A possible reason for this difference lies in the effects on adult life span (Figure 2b). Haplotype C was not significantly associated with adult life span in either sex, whereas haplotype D was associated with adult female life span and the point estimates of haplotype D for male adult life span were in the same direction though not significant. If individuals carrying haplotype D remain in the population longer, as this suggests, then they contribute to the significant increase in haplotype frequency in the standing population each year. To our knowledge, our study is the first demonstrating that the frequency of a MHC haplotype conferring selective advantage also increased through the study period in a wild population. In addition, we found the cumulative frequency change of two haplotypes was more even than expected, such results demonstrat balancing selection may act on those haplotypes to maintain the frequency around the equilibrium.

Several aspects of our analysis improve on earlier MHC‐fitness association studies. Benefiting from MHC haplotype data generated by locus‐specific genotyping, this is one of the few studies using dosage models to precisely examine whether there is evidence for heterozygote advantage and divergent allele advantage which are caused by non‐additive genetic effects (Hu et al., 2015; Lenz et al., 2015). Indeed, we demonstrated a signature of divergent haplotype advantage in juvenile survival. In addition, we used animal models to study the association between MHC and fitness measurements in wild populations. As the animal model accounts for relatedness, we predicted that it would be more conservative and show fewer associations than GLMMs that did not account for relatedness. In fact, we found the results to be largely consistent between the two models. This may be due to the generally low heritability of the fitness traits (Table S13). However, for traits which have a higher heritability, the results of animal models will be more conservative as they would exclude the false‐positive effects caused by genomic regions other than the MHC.

Long‐term individual‐based studies like the Soay sheep project also enable repeat measurements of the strength and direction of selection over time (Siepielski et al., 2009, 2011). In a previous study, three MHC‐linked microsatellite alleles were found to be associated with survival in Soay sheep using individuals from 1985 to 1994 (Paterson et al., 1998). Microsatellite alleles OLADRB 257 and 205 were associated with decreased juvenile and yearling survival, respectively, whereas the OLADRB 263 allele was associated with increased yearling survival. Some microsatellite alleles can be linked directly to the MHC class II haplotypes studied here: OLADRB allele 257 is linked to haplotype B (Table S14). Thus, we might expect some consistency between the results from the previous study and the current one. However, in our analysis of juvenile survival, we did not find any evidence for haplotype‐specific effects (Wald tests not significant; Table 2). The inconsistency between the previous and current study is probably due to differences in genotyping method, sample size and statistical methods including fitting relatedness and inbreeding. In addition, alleles OLADRB 205 and 263 are associated with multiple haplotypes, and we have studied annual survival rather than yearling survival, which may also contribute to a failure to repeat the results.

In summary, we used well‐characterised MHC class II haplotypes to investigate MHC‐linked fitness effects in Soay sheep. To our knowledge, our data set, with more than 3000 individuals, is the largest yet used to study the selection on MHC variation in a wild population. Our results support the existence of contemporary selection on MHC class II variation in Soay sheep, when genetic relatedness and inbreeding are controlled for. We observed that the frequency of a rare MHC haplotype with a selective advantage has increased significantly during the study period. Our study highlights the importance of investigating selection on MHC genes across the whole lifespan and frequency change of MHC haplotypes or alleles.

## CONFLICT OF INTEREST

The authors declare that they have no competing interests.

## AUTHORSHIP

W.H, J.D.H and J.M.P conceived the study. K.L.D generated MHC data with the help from S.E.J and K.T.B. W.H performed data analysis with the help from J.D.H and S.E.J. W.H wrote the manuscript with editorial input from all the other authors.

### PEER REVIEW

The peer review history for this article is available at https://publons.com/publon/10.1111/ele.13957.

### OPEN RESEARCH BADGES

This article has earned an Open Data badge for making publicly available the components of the research methodology needed to reproduce the reported procedure and analysis. All materials are available at: https://figshare.com/articles/dataset/Selection_on_MHC_genes_in_Soay_sheep/14498295/1. DOI: https://doi.org/10.6084/m9.figshare.14498295.v1.

## Supporting information

Supplementary Material

## Data Availability

Data and code used in this paper are available from the following link: https://doi.org/10.6084/m9.figshare.14498295.v1.
